# Adequate Care Coordination is Associated with Reduced Unmet Mental Health Needs for Children

**DOI:** 10.1007/s10560-026-01101-w

**Published:** 2026-03-24

**Authors:** Genevieve Graaf, Kristin Gigli, Phillip Hughes, Kathleen C. Thomas

**Affiliations:** 1 School of Social Work, The University of Texas at Arlington, Arlington, USA; 2 College of Nursing and Health Sciences, The University of Texas at Arlington, Arlington, USA; 3 School of Pharmacy and Pharmaceutical Sciences, Department of Pharmaceutical Sciences, Binghamton University, Johnson City, NY, USA; 4 Division of Pharmaceutical Outcomes and Policy, Eshelman School of Pharmacy, University of North Carolina at Chapel Hill, Chapel Hill, USA; 5 Cecil G. Sheps Center for Health Services Research, University of North Carolina at Chapel Hill, Chapel Hill, USA

**Keywords:** Child mental health, Youth mental health, Care coordination, School mental health

## Abstract

Children with special healthcare needs (CSHCN), especially those with mental health needs, often receive services from multiple service sectors, including schools. Thus, care coordination may play a critical role in access to mental health care for these children. This cross-sectional study used multivariable logistic regression with pooled data from the National Survey of Children’s Health (2020–2023), focusing on a subsample of children with caregiver-reported mental health problems and a reported need for mental healthcare. We estimated the prevalence of parent-reported need for and receipt of effective care coordination and provider communication with schools. It also measures the association between parent-reported receipt of these services with unmet need for mental healthcare. Most caregivers reported need for care coordination (51.3%) and provider communication with schools (60.7%). Among those with need, 48.3% reported not having their care coordination needs met, and 57.2% of caregivers reported unmet need for provider communication with schools. The probability of reporting unmet mental health needs was 12percentage points lower when children had all needs for care coordination met and 10 points lower when providers communicated with schools. Results suggest that having care coordination needs met and provider communication with schools may play an important role in children accessing needed mental healthcare.

**Supplementary Information:**

The online version contains supplementary material available at 10.1007/s10560-026-01101-w.

## Introduction

Approximately 30% of children with special health care needs (CSHCN) have some combination of medical needs, emotional or behavioral health needs, or developmental disorders, while almost 20% have only emotional, behavioral, or developmental health needs (Cheak-Zamora & Thullen, 2017). Children with special health care needs have poorer academic outcomes than their peers: they have lower scores on a range of academic performance indicators (Forrest et al., 2011), lower rates of on-time high school completion (Mckinley Yoder & Cantrell, 2019), and miss more school (Houtrow et al., 2012) than non-CSHCN. CSHCN face additional barriers—such as difficulty participating fully in classroom activities, reduced motivation, disruptive behavior, and experiences of bullying—that undermine their engagement and sense of safety at school. These challenges are especially pronounced for those with emotional, behavioral, or developmental needs (Forrest et al., 2011; Bethell et al., 2012).

Expanding access to mental health services has been shown to help address these academic disparities. For CSHCN with emotional or behavioral conditions or developmental challenges, receiving appropriate mental health care is associated with better school enrollment and attendance patterns, improved retention, and fewer disciplinary actions and self-harm incidents (Golberstein et al., 2024; Kang-Yi et al., 2018, 2024). However, many of the behavioral health needs of these children go unmet. Over 20% of all children with caregiver-reported mental health problems and need for mental health care report unmet mental health need, and 46% of these families reported some level of difficulty in accessing mental health care (Graaf et al., 2025). Providing mental health care through schools, which reduces scheduling and transportation barriers for families, has become a widely promoted solution to addressing high rates of unmet mental health needs in children (Hoover et al., 2020). As such, schools now serve as primary settings for recognizing students’ behavioral health needs, initiating referrals, and frequently providing related services (Duong et al., 2020; Hoover et al., 2020).

### CSHCN and Complex Service Systems

CSHCN, especially those with mental health needs, also often receive care from multiple service sectors (e.g., public health, schools, health systems) to address their complex health or behavioral health concerns (Hansen et al., 2004; Kuo et al., 2018). Families who receive professional assistance coordinating care across providers are more likely to access needed specialist care (Miller, 2014), assistive technologies, and specialized therapies (Graaf & Gigli, 2022) than those who do not. For this reason, assistance in care coordination across services is highly valued by caregivers (Graaf et al., 2024) and can play a critical role in accessing adequate care for these children (Lindly et al., 2020).

A key component of care coordination for children with complex health care needs is coordination and collaboration between school systems and health and behavioral health care providers (Kuo et al., 2018). Caregivers of children with mental health needs frequently cite the importance of provider communication and cooperation with their children’s schools (Graaf et al., 2024), where their children spend the majority of their time and where classroom accommodations and school-based supports are particularly important for their educational success. Evidence suggests that receipt of adequate care coordination, including provider communication with schools, is associated with reduced unmet health care needs, reduced use of emergency room care (Chowdhury et al., 2026), and increased access to specialized medical care (Graaf & Gigli, 2022; Miller, 2014). One study assessing the relationship between receipt of care within a medical home and mental health care access among children with autism spectrum disorder (ASD), examined this relationship across all subcomponents of the medical home measure, including receipt of effective care coordination. Caregivers of children with ASD who reported ineffective care coordination also reported more difficulty accessing mental health care and higher rates of unmet need for mental health care than counterparts reporting effective care coordination (Ezeh et al., 2022).

Though receipt of care coordination is demonstrated to be associated with reduction in a range of unmet health care needs, compared to children with other types of complex healthcare needs, children with mental health conditions are less likely to receive adequate care coordination (Miller et al., 2018). Further, little is known about the prevalence of care coordination for children with mental health conditions nationwide or the role it may play in access to mental health care. A lone study, drawing from 2007 data, demonstrated that over 40% of children with mental health conditions had unmet need for care coordination (Brown et al., 2014).

Even less is known about a subcomponent of care coordination highly valued by caregivers: provider/school communication (Graaf et al., 2024). In a national study of the general child population, 23.5% of caregivers reported that health care providers communicated with their child’s school, daycare or special education program (Geffel et al., 2022). Parents of children with mental health conditions were more likely to report provider/school communication than children in the general population. However, caregiver-reported provider/school communication was less common for children with mental health conditions than for children with other types of physical health or medical conditions (Geffel et al., 2022).

Despite Ample research demonstrating that care coordination is associated with reductions in unmet needs for a range of health care services for CSHCN, but there are few nationally representative estimates of caregiver need and unmet need for help in arranging care for children with mental health conditions. Further, no study has examined associations between the receipt of care coordination, or its important subcomponent provider/school communication, in relation to access to mental health care for children with mental health conditions. To address this gap, this study draws on updated nationally representative data (2020–2023) to examine this relationship for children with caregiver-reported mental health problems and need for mental healthcare. Specifically, this study aims to (1) assess the prevalence of caregiver-reported need for—and receipt of—adequate care coordination and provider communication with schools and (2) estimate the association between caregiver-reported receipt of these services and unmet need for mental healthcare.

## Methods

We conducted a cross-sectional study, pooling data from the National Survey of Children’s Health (NSCH; 2020–2023). Information about the NSCH methodology is detailed elsewhere (Ghandour et al., 2018). The sample included children (age 0–17) with caregiver-reported mental health problems and need for mental health care. Mental health problems were identified through either a caregiver-reported child emotional, behavioral, or developmental problem on the Children with Special Health Care Needs Screener subscale within the NSCH, or a caregiver’s report of their child’s receipt of a mental health diagnosis (anxiety, depression, attention-deficit hyperactivity disorder, or behavioral or conduct problems) (Bethell et al., 2015). To identify children with caregiver-reported need for mental health care, the following survey question was used: “During the past 12 months, has this child received any treatment or counseling from a mental health professional? Mental health professionals include psychiatrists, psychologists, psychiatric nurses, and clinical social workers.” Children whose caregiver reported “Yes” or “No, but this child needed to see a mental health professional” were coded as having need for mental health care. Children whose caregiver responded, “No, this child did not need to see a mental health professional,” were excluded from the sample as they indicated no need for mental health care (See Fig. 1 in the online Supplement). The outcome variable, unmet need for mental health care, was also constructed from this variable. Consistent with several other studies, children with mental health problems whose caregivers reported that they needed to see a mental health professional in the past 12 months but did not see one were coded as having unmet mental health need (Casseus, 2023; Lebrun-Harris et al., 2019; Pasli & Tumin, 2022).

Analysis addressed two predictors of unmet mental health need: (1) having care coordination needs met, and (2) provider communication with a child’s school or childcare. The NSCH includes a six-item measure for Effective Care Coordination, which assesses if a family (1) received assistance with care coordination *or* (2) needed extra help with coordinating care. If they needed extra help with care coordination, they were asked additional questions about how often they received all the extra help they needed and if their child’s doctors communicated with other providers or with their child’s school or other educational programs. Using this measure, need and unmet need for care coordination were structured according to methods developed by Brown and colleagues (see Fig. 2 in the online supplement) (Brown et al., 2014).

Caregivers who reported that their child saw more than one type of health care provider in the past year were asked the following two questions: “During the past 12 months, have you felt you could have used extra help arranging or coordinating this child’s care among the different healthcare providers or services?”; and “During the past 12 months, how often did you get as much help as you wanted with arranging or coordinating care?” We created two binary variables to measure care coordination need and unmet need (See Fig. 1). Caregivers reporting that no one helped them arrange or coordinate their child’s care and that they did not need extra help were categorized as having no need for care coordination. Having all care coordination needs met was defined as either (1) receiving help arranging or coordinating care and not needing any extra help, or (2) needing extra help and usually or always receiving as much extra help as they wanted. Unmet need for care coordination was defined as sometimes or never receiving as much help in arranging or coordinating care as was wanted.

Provider communication with schools was drawn from a subitem in the Effective Care Coordination measure. It is derived from a question asking caregivers, for all children, regardless of how many healthcare providers or doctors they had seen in the past 12 months, if their child’s healthcare provider had communicated with their child’s school, childcare provider, or special education program. Children whose caregivers reported “yes” were coded as needing provider communication with schools and as having received that service. Due to healthcare privacy laws, a caregiver would have to provide written and signed authorization for the provider to communicate with their child’s school. For this reason, our analysis assumed that provider/school communication would likely not have taken place unless a caregiver believed it was needed and subsequently authorized it. Children whose caregivers responded “Did not need health care provider to communicate with these providers” were coded as not needing provider communication with schools. Children whose caregivers reported “no” were coded as needing provider communication with their child’s school but not receiving it (unmet need). The rationale for this coding is that these children’s caregivers failed to select the answer option “Did not need health care provider to communicate with these providers”, indicating perceived need.

**Analysis.** Univariate analysis described rates of caregiver-reported unmet need for mental healthcare and need and unmet need for care coordination and provider communication with schools or childcare. Separate logistic regression models estimated associations between each predictor variable and parent-reported unmet mental health need, each using separate subsamples. For care coordination, only children whose caregiver reported that the child saw more than one type of provider in the last year were included in the subsample. For provider communication with schools, only those whose caregiver reported need for providers to communicate with schools were included in the subsample. Regression analyses controlled for child and family covariates, drawn from the same dataset, guided by the Behavioral Model of Health Service Use (Andersen, 1995). Low missingness supported a complete-case approach for analysis, (Langkamp et al., 2010) resulting in slightly smaller sample sizes after inclusion of covariates.

Calculation of odds ratios incorporates unobserved study and modeling error into the denominator of the calculation, making odds ratios relatively ungeneralizable (Norton et al., 2024). For this reason, average marginal effects, which are more transferrable across study designs, were generated in marginal post-estimation for each predictor variable (Norton et al., 2024). Analyses were conducted using STATA v.16.1 (StataCorp, College Station, TX), employing the survey (*‘svy’* and *‘subpop’*) commands to account for survey weighting, stratification, and clustering to generate nationally representative results.

Data used in this study spanned the COVID-19 pandemic which created changes in children’s need for mental health care and practices in mental health service delivery (Kauhanen et al., 2023; Palinkas et al., 2021; Purtle et al., 2022; Sklar et al., 2021; Zablotsky et al., 2022), resulting in increased unmet mental health need for some children (Gigli & Graaf, 2022) and increased barriers to accessing mental health care (, Graaf, et al., Under review). To assess if pandemic impacts were observable in our data and would thus contribute to inaccurate assessments of usual care circumstances, sensitivity analyses were conducted using the National Survey of Children’s Health pooling data from 2016 to 2019. Because results were similar to those generated using 2020–2023 data (See Exhibit A in the online Supplement), results from analysis with the most recent data are reported here.

## Results

Weighted proportions of need and unmet need for mental health care, care coordination, and provider communication with schools or childcare are reported in Table 1. For children with caregiver-reported mental health problems and need for mental healthcare, 18.6% reported unmet mental health need. Half (51.3%) reported a need for care coordination and 60.7% reported a need for providers to communicate with their child’s school or childcare. Almost half of caregivers needing care coordination (48.3%) reported not having all their care coordination needs met, with 57.2% of families needing provider communication with schools reporting that providers failed to do so. In logistic regression (see Table 2), the probability of reporting unmet mental health needs was 12% points lower when caregivers had all their care coordination needs met [Average Marginal Effect (AME) = −0.12 (−0.16, −0.08)] and 10% points lower when providers communicated with school or childcare [AME = −0.10 (−0.13, −0.07)]. See the online supplement (Exhibit B and C) for sample description and full models).

## Discussion

Among children with mental health problems, caregiver-reported need for care coordination and provider/school communication is high. However, most families needing these services did not receive an adequate amount of care coordination and experienced inadequate provider communication with schools. Results suggest that effective care coordination and provider communication with schools may play an important role in ensuring these children access needed mental health services. In this study, these activities were associated with reduced probability of reporting unmet mental health needs. Improved access to needed mental healthcare may be facilitated by coordination and communication across service sectors. 2024).

Given rising concerns about children’s increasing need for mental health care (Foy et al., 2019), study results may have important implications for pediatric primary care, specialist practices, and school- and community-based mental health providers. Families are often unaware of where to access mental health care and schools can be important in connecting children with mental health services, either by providing them directly in the school settings or referring families to community-based supports (Hoover et al., 2020). The American Academy of Pediatrics reaffirmed the Mental Health Competencies for Pediatric Practice in 2025, which emphasizes the importance of pediatricians engaging a child’s schools to appropriately address their mental health needs (Foy et al., 2019). Provider communication with schools or childcare can alert school personnel to the need for care, provide a pathway for schools to alert the pediatrician to mental health needs, and can support the coordination of that care to ensure that schools are fully informed about medication use or behavioral or emotional support plans.

Many challenges to adequately supporting care coordination and school communication exist in pediatric health care or community mental health practices. These include inadequate reimbursement for care coordination activities, weak partnerships or connections across service sectors, inadequate mental health workforce in the community or schools, and insufficient pediatric care staffing and expertise to address more complex mental health needs (Foy et al., 2019; National Academies of Sciences, Engineering, and Medicine, 2024). However, structured and routine communication processes, such as regularly scheduled care team meetings or check-ins and the support of healthcare social workers, can assist in overcoming some of these challenges (Ross et al., 2019).

A growing body of research highlights that structural, cross-system frameworks, such as the Interconnected Systems Framework (ISF) and High-Fidelity Wraparound, offer concrete strategies for improving coordination between schools, families, and community mental health providers. ISF directly addresses gaps in cross-sector communication by establishing team structures, routine communication processes, and interorganizational supports tailored to specific levels of youth behavioral health complexity and severity (Huber et al., 2016). For children with more complex behavioral health needs, Wraparound provides an additional, well-studied model of care coordination that is individualized, family-centered, and team-based. Fidelity to Wraparound principles has been associated with improvements in behavioral and functional outcomes, family satisfaction, and reductions in restrictive placements, demonstrating that structured, collaborative coordination processes can meaningfully improve outcomes for youth with serious emotional and behavioral challenges (Bruns et al., 2005; Olson et al., 2021). 

Social workers based in schools, healthcare settings, or community mental health centers play a central role in these coordinated, multi-system models by serving as a conduit between families, schools, and community mental health systems. In ISF and Wraparound, social workers frequently participate on child and family care teams, ensure fidelity to family-centered planning, facilitate communication across sectors, and help to establish and implement multi-stakeholder care plans. All of these activities are essential for effective cross-system collaboration and consistent care planning and implementation (Bruns et al., 2008; Kuo et al., 2018).

To support these efforts in pediatric practices and other community-based behavioral health settings, attention to increasing time and compensation for care coordination activities is needed (National Academies of Sciences, Engineering, and Medicine, 2023, 2024). State mental health agencies, Medicaid authorities, and insurance market governing bodies can play an important role in the extent to which these services are covered for children in their state and the adequacy of reimbursement rates for these services (Graaf & Snowden, 2021). While many states provide financing for care coordination and Wraparound for children with complex behavioral health needs (Graaf & Snowden, 2017), results suggest that children with less severe mental health conditions may also benefit from these types of services. Variation in geographic availability is also an on-going concern (Graaf et al., 2025). Public concern about growing mental health need may be leveraged to encourage state administrations to expand coverage and increase payment for care coordination activities for all children with behavioral health needs. Such policy actions may play a pivotal role in expanding access to needed mental health care for children.

## Limitations

These findings are not without limitations. First, the NSCH excludes children in institutional settings, including justice or psychiatric institutions, among whom more complex mental health needs are overrepresented. Further, the study is limited by the data collected in the NSCH, as well as the way in which certain constructs were measured in the NSCH. Covariates were limited to individual characteristics and factors included in the dataset, and measures were based on caregiver self-report. Thus, indicators or mental health problems or use of mental health services were not assessed using validated clinical measures which, combined with recall bias, may result in inaccurate reports of unmet mental health need (Hoagwood et al., 2000). Further, the measure of unmet need was based on caregivers’ report regarding whether their child saw a mental health provider in the last twelve months. This measure may exclude (1) children whose mental health needs were met by their primary care provider and (2) children who saw a mental health provider but whose mental health needs were not fully met. Finally, the measure of provider/school communication asks if the child’s “health care provider” communicated with the school, daycare or special education program. While the term “health care provider” may include behavioral health professionals, it may also be that a child’s primary care doctor or specialist communicated with the child’s school but a behavioral health provider did not.

## Conclusion

This study demonstrates that effective care coordination and provider communication with schools are associated with reduced unmet mental health needs among children with caregiver-reported mental health concerns. These results reinforce longstanding claims that cross-system collaboration can meaningfully strengthen service access and continuity for children and youth (Larson, et al., 2022). However, despite the availability of several empirically supported models of coordinating care for children and youth with behavioral health needs, national data in this study suggest that many families continue to experience gaps in coordination and communication, underscoring the need for broader and more consistent implementation. By highlighting the importance of these coordination activities at a population level, this study points to actionable policy and practice opportunities to expand care coordination efforts that integrate care across service sectors to better meet the needs of children with mental health conditions and their families.

**Table 1:** Mental Health Care and Care Coordination Among Children with Mental Health Problems & Need for Mental Health Care (*n* = 22,509)

	*n*	%*
*Unmet Need for Mental Health Care*		
No unmet need mental health care	18,892	81.4%
Unmet need mental health care	3,617	18.6%
*Need for Care Coordination*,* among children seeing 2 + healthcare providers* (*n* = 17,236)		
Had need for care coordination	8,732	51.3%
No need for care coordination	8,360	48.7%
*Unmet Need Care Coordination*, ** among children seeing 2 + healthcare providers (*n* = 17,236)		
Unmet need for care coordination	3,770	48.3%
No unmet need for care coordination	4,242	51.7%
*Need for Doctor Communication with Schools and Childcare*		
No school communication needed	8,499	39.3%
Doctor/school communication needed	12,148	60.7%
Doctor Communicates with Schools and Childcare**		
Doctors did not communicate with school or childcare	6,929	57.2%
Doctors communicate with child’s school or childcare	5,219	42.8%

Data Source: National Survey of Children’s Health (NSCH), 2020–2023; *Weighted Proportions; ** Among those with need for care coordination or provider communication with schools or childcare

Alternative text: 18.6% of children and youth with parent-reported mental health problems and need for mental healthcare reported unmet mental health need. Over half of caregivers reported need for care coordination, with just under half of families having all their care coordination needs met. Over 60% of families reported need for providers to communicate with their child’s school or childcare but almost 60% of these families reported that providers failed to do so

**Table 2:** Association between Care Coordination and Mental Health Service Use among Children with Mental Health Problems and Need for Mental Health Care

	AME	*p*<	95%	
Care Coordination Need Met**n = 7,804*	−0.12	< 0.001	−0.16	−0.08
Provider Communicated with Schools or Daycare *n* = 11,796*	−0.10	< 0.001	−0.13	−0.07

Data Source: National Survey of Children’s Health (NSCH), 2020–2023; *complete cases; ** Among those with need for care coordination; Models controlled for child sex, race/ethnicity, age, impairments in daily activities, insurance type, gaps in insurance, family poverty level, family structure, household language, highest caregiver education level, and survey year; AME = Average Marginal Effect

Alternative text: The probability of unmet mental health needs was 8% points lower when children received effective care coordination and 11% points lower when providers communicated with school or childcare

## Supplementary Information

Below is the link to the electronic supplementary material.

Supplementary Material 1

## Data Availability

Data is available publicly through the Data Resource Center for Child and Adolescent Health at https://www.childhealthdata.org/learn-about-the-nsch/NSCH.
