# Supplementary material for: Adequate Care Coordination is Associated with Reduced Unmet Mental Health Needs for Children
Source: Child Adolesc Social Work J. 2026 Mar 24;43(4):1295–302. doi: 10.1007/s10560-026-01101-w (PMC13427786; doi:10.1007/s10560-026-01101-w)
Supplement: Supplementary file 1 — Supplementary Material 1 [file 10560_2026_1101_MOESM1_ESM.docx]

**Figure 1. Children with Mental Health Problems and Need for Mental Health Care: Study Sampling and Sample Sizes**

Children in the NSCH, 2020-2023

(n = 202,934)

**EXCLUDED:** No Mental Health Problem (n = 133,221)

**Children with Mental Health Problems**

(assessed adapting Bethell et al.’s methods^9^)

(n = 41,124)

“Yes” (**No Unmet Need**)

(n=18,892)

or

“No, but this child needed to see a mental health professional.” (**Unmet Need)**

(n=3,617)

**Total Children with Mental Health Problems and Need for Mental Health Care**

**(n= 22,509)**

**Need for Mental Health Care**

“During the past 12 months, has this child received any treatment or counseling from a mental health professional? Mental health professionals include psychiatrists, psychologists, psychiatric nurses, and clinical social workers.”

**EXCLUDED:** “No, this child did not need to see a mental health professional” (n= 18,418)

**Figure 2. Care Coordination and Provider/School Communication Need and Unmet Need: Study Sampling and Sample Sizes**

“During the past 12 months, did anyone help you arrange or coordinate this child’s care among the different doctors or services that this child uses?”

During the past 12 months, how often did you get as much help as you wanted with arranging or coordinating this child’s health care?

**Need for Provider Communication with Schools and Daycare**

During the past 12 months, did this child’s health care provider communicate with the child’s school, childcare provider, or special education program?

**EXCLUDED:** "Did not see more than one health care provider in past 12 months" (n=5,273)

Yes (n=6,086)

During the past 12 months, have you felt that you could have used extra help arranging or coordinating this child’s care among the different health care providers or services?

**UNMET NEED for Care Coordination**

(n=3,770)

**Total Children with Mental Health Problems and Need for Mental Health Care**

**(n= 22,509)**

Usually/Always

**(**n**=793)**

Yes (n=4,636)

No (n=12,489)

No (n=11,150)

Yes (n=5,219)

**Provider/School Communication Need Met**

No (n=6,929)

**Provider/School Communication Need Not Met**

**No Need for Provider/School Communication** (n=8,499)

No (n=12,489)

Sometimes or Never (n=3,770)

**MET NEED for Care Coordination**

(n=4,242)

**NO NEED for Care Coordination**

(n=8,360)

| **Exhibit A. Association between Care Coordination and Mental Health Service Use among Children with Mental Health Problems and Need for Mental Health Care, 2016-2019** | | | | |
| --- | --- | --- | --- | --- |
|  | **AME** | **p<** | **95%** | |
| **Care Coordination Need Met****  n=3,836* | -0.10 | <0.001 | -0.14 | -0.06 |
| **Provider Communicated with Schools or Daycare**  n=7,332* | -0.11 | <0.001 | -0.15 | -0.07 |

Data Source: National Survey of Children’s Health (NSCH), 2016-2019; *complete cases; ** Among those with need for care coordination; Models controlled for child sex, race/ethnicity, age, impairments in daily activities, insurance type, gaps in insurance, family poverty level, family structure, household language, highest caregiver education level, and survey year; AME = Average Marginal Effect

| **Exhibit B. Sample Description: Children with Mental Health Problems & Need for Mental Health Care (n= 22,509)** | | |
| --- | --- | --- |
|  |  |  |
|  | **n** | **%*** |
| **Child Sex** |  |  |
| Male | 11,352 | 51.0% |
| Female | 11,157 | 49.0% |
| **Child Race/Ethnicity** |  |  |
| White | 15,627 | 54.5% |
| Black, non-Hispanic | 1,328 | 12.9% |
| Hispanic | 3,042 | 23.1% |
| Other/Multiracial | 2,512 | 9.5% |
| **Child Age** |  |  |
| 0-5 years | 1,573 | 6.5% |
| 6-11 years | 7,229 | 35.3% |
| 12-17 years | 13,707 | 58.2% |
| **Household Income** |  |  |
| Below 200% FPL | 7,078 | 39.9% |
| 200% FPL and above | 15,431 | 60.1% |
| **Insurance Type** |  |  |
| Private only | 13,206 | 50.9% |
| Public only | 6,549 | 36.1% |
| Public and Private | 1,804 | 8.5% |
| Not Insured | 647 | 4.4% |
| **Insured Continuously for prior 12 months** |  |  |
| No | 981 | 5.8% |
| Yes | 21,412 | 94.2% |
| **Source of Usual Care** |  |  |
| No | 809 | 5.3% |
| Yes | 21,607 | 94.7% |
| **Complexity of Needs** |  |  |
| Less Complex Needs | 9,148 | 41.4% |
| More Complex Needs | 13,361 | 58.6% |
| **Parent Education Level** |  |  |
| Less than High School | 455 | 7.0% |
| High School graduate | 2,913 | 18.3% |
| More than High School | 19,141 | 74.7% |
| **Family Structure** |  |  |
| Two parents | 13,029 | 52.9% |
| Other Family Type | 9,374 | 47.1% |
| **Household Language** |  |  |
| English | 21,552 | 92.3% |
| Not English | 822 | 7.7% |
| **Year** |  |  |
| 2020 | 4,616 | 23.4% |
| 2021 | 5,233 | 23.8% |
| 2022 | 6,307 | 26.3% |
| 2023 | 6,353 | 26.4% |
| Data Source: National Survey of Children’s Health (NSCH), 2016-2022; *Weighted Proportions  Alternative Text: The sample is predominantly comprised of white males aged 12-17. Most of these children reside in households with incomes above 200% of the FPL, have private insurance, and are continuously insured with a usual source of care. The data also highlights that a majority of these children have more complex needs, and that English is the primary household language. | | |

| **Exhibit C. Association between Care Coordination and Unmet Mental Health Needs among Children with Mental Health Problems and Need for Mental Health Care** | | | | | | | |  |  |  |
| --- | --- | --- | --- | --- | --- | --- | --- | --- | --- | --- |
|  | **Care Coordination Need Met** n=7,804*** | | |  | **Doctor Communicates with Schools or Daycare n=11,796*** | | |  |  |  |
|  | **OR** | **95%** | |  | **OR** | **95%** | |  |  |  |
| **Predictor of Unmet Mental Health Need** | 0.44 | 0.34 | 0.58 |  | 0.49 | 0.39 | 0.61 |  |  |  |
| **Child Sex** | 0.79 | 0.61 | 1.03 |  | 0.69 | 0.56 | 0.85 |  |  |  |
|  |  |  |  |  |  |  |  |  |  |  |
| **Child Race/Ethnicity** |  |  |  |  |  |  |  |  |  |  |
| Black, non-Hispanic | 0.98 | 0.64 | 1.50 |  | 1.07 | 0.76 | 1.49 |  |  |  |
| Hispanic | 1.24 | 0.88 | 1.73 |  | 0.67 | 0.50 | 0.91 |  |  |  |
| Other/Multiracial | 0.98 | 0.69 | 1.40 |  | 1.04 | 0.71 | 1.51 |  |  |  |
|  |  |  |  |  |  |  |  |  |  |  |
| **Child Age** |  |  |  |  |  |  |  |  |  |  |
| 6-11 years | 0.60 | 0.41 | 0.88 |  | 0.70 | 0.48 | 1.01 |  |  |  |
| 12-17 years | 0.45 | 0.31 | 0.66 |  | 0.45 | 0.31 | 0.64 |  |  |  |
|  |  |  |  |  |  |  |  |  |  |  |
| **Household Income: Above 200% of FPL** | 1.01 | 0.75 | 1.37 |  | 0.80 | 0.62 | 1.04 |  |  |  |
|  |  |  |  |  |  |  |  |  |  |  |
| **Insurance Type** |  |  |  |  |  |  |  |  |  |  |
| Public | 1.11 | 0.81 | 1.52 |  | 1.29 | 0.98 | 1.70 |  |  |  |
| Public and Private | 1.05 | 0.71 | 1.55 |  | 1.14 | 0.74 | 1.76 |  |  |  |
| Not Insured | 1.34 | 0.49 | 3.66 |  | 1.17 | 0.55 | 2.49 |  |  |  |
|  |  |  |  |  |  |  |  |  |  |  |
| **Insured Continuously for Prior 12 Months** | 0.62 | 0.34 | 1.14 |  | 0.53 | 0.32 | 0.89 |  |  |  |
| **Has a Source of Usual Care** | 0.64 | 0.29 | 1.41 |  | 0.53 | 0.32 | 0.90 |  |  |  |
|  |  |  |  |  |  |  |  |  |  |  |
| **Complexity of Needs** |  |  |  |  |  |  |  |  |  |  |
| More Complex Needs | 0.86 | 0.65 | 1.13 |  | 1.00 | 0.80 | 1.24 |  |  |  |
|  |  |  |  |  |  |  |  |  |  |  |
| **Parent Education Level** |  |  |  |  |  |  |  |  |  |  |
| High School | 1.10 | 0.46 | 2.62 |  | 1.35 | 0.72 | 2.51 |  |  |  |
| More than High School | 1.49 | 0.68 | 3.25 |  | 1.73 | 0.96 | 3.12 |  |  |  |
|  |  |  |  |  |  |  |  |  |  |  |
| **Family Structure - Two parent family** | 1.40 | 1.08 | 1.82 |  | 1.01 | 0.81 | 1.27 |  |  |  |
| **Household Language - Not English** | 0.90 | 0.47 | 1.70 |  | 1.59 | 1.00 | 2.52 |  |  |  |
| **Year** | 1.15 | 1.03 | 1.29 |  | 1.03 | 0.92 | 1.14 |  |  |  |
| Data Source: National Survey of Children’s Health (NSCH), 2020-2023; *complete cases; ** Among those with need for care coordination  Alternative Text: For both "Care Coordination Needs Met" and "Doctor Communicates with Schools or Daycare," an Odds Ratio (OR) of less than 1 for "Predictor of Unmet Mental Health Need" suggests that having care coordination needs met is associated with a lower likelihood of unmet mental health needs. | | | | | | | |  |  |  |
